# Seroprevalence of dengue virus in two districts of Kaohsiung City after the largest dengue outbreak in Taiwan since World War II

**DOI:** 10.1371/journal.pntd.0006879

**Published:** 2018-10-24

**Authors:** Jih-Jin Tsai, Ching-Kuan Liu, Wen-Yang Tsai, Li-Teh Liu, Jasmine Tyson, Ching-Yi Tsai, Ping-Chang Lin, Wei-Kung Wang

**Affiliations:** 1 Tropical Medicine Center, Kaohsiung Medical University Hospital, Kaohsiung, Taiwan; 2 Division of Infectious Diseases, Department of Internal Medicine, Kaohsiung Medical University Hospital, Kaohsiung, Taiwan; 3 Center for Dengue Fever Control and Research, Kaohsiung Medical University, Kaohsiung, Taiwan; 4 School of Medicine, College of Medicine, Kaohsiung Medical University, Kaohsiung, Taiwan; 5 Graduate Institute of Medicine, College of Medicine, Kaohsiung Medical University, Kaohsiung, Taiwan; 6 Department of Neurology, Kaohsiung Medical University Hospital, Kaohsiung, Taiwan; 7 Department of Tropical Medicine, Medical Microbiology and Pharmacology, John A. Burns School of Medicine, University of Hawaii at Manoa, Honolulu, Hawaii, United States of America; 8 Department of Medical Laboratory Science and Biotechnology, College of Medicine and Life Science, Chung-Hwa University of Medical Technology, Tainan, Taiwan; University of California San Francisco, UNITED STATES

## Abstract

Dengue virus (DENV) is the leading cause of arboviral diseases in humans worldwide. In this study, we investigated the seroprevalence of DENV infection in two districts of Kaohsiung City, a metropolis in southern Taiwan, where major dengue outbreaks have occurred in the past three decades. We enrolled 1,088 participants from the Sanmin and Nanzih districts after the dengue outbreak of 2015, the largest in Taiwan since World War II, and found an overall DENV seroprevalence of 12.4% (95% confidence interval: 10.5–13.4%) based on the InBios DENV IgG ELISA kit. The ratios of clinically inapparent to symptomatic infections were 2.86 and 4.76 in Sanmin and Nanzih districts, respectively. Consistent with higher case numbers during recent outbreaks, the DENV seroprevalence was higher in Sanmin district (16.4%) than in Nanzih district (6.9%), suggesting district differences in seroprevalence and highlighting the importance of screening the DENV immune status of each individual before using the currently available DENV vaccine, Dengvaxia. In the two districts, the seroprevalence rates increased from 2.1% (in the 30–39-year age group) to 17.1% (60–69) and 50% (70–79). The pattern of a sharp and significant increase in seroprevalence in the 70–79-year age group correlated with a dramatic increase in the proportion of clinically severe DENV infections among total dengue cases in that age group. This differed from observations in the Americas and Southeast Asia and suggested that a large proportion of monotypically immune individuals together with other risk factors may contribute to clinically severe dengue among the elderly in Taiwan.

## Author summary

Dengue virus (DENV) is the most important cause of mosquito-borne viral disease in humans worldwide. Investigating DENV seroprevalence in Kaohsiung City after the largest dengue outbreak in Taiwan since World War II, we found an overall seroprevalence of 12.4% and heterogeneity in seroprevalence within a metropolis; this together with the low efficacy and potential disease enhancement of the currently available dengue vaccine (Dengvaxia) in DENV-naive individuals highlighted the importance of checking the DENV immune status of individuals prior to Dengvaxia vaccination. The pattern of a sharp and significant increase in DENV seroprevalence in the 70–79 year age group in Kaohsiung City was different from that in the Americas and Southeast Asia, and suggested that a large proportion of monotypically immune individuals in that age group together with other risk factors may contribute to clinically severe dengue disease among the elderly in Taiwan.

## Introduction

The four serotypes of dengue virus (DENV1-DENV4), which belongs to the genus *Flavivirus* of the family *Flaviviridae*, cause the most important mosquito-borne viral disease in humans in the tropical and subtropical regions [1,2]. It has been estimated that more than 3 billion people living in more than 120 countries are at risk of DENV infection and approximately 390 million DENV infections occur annually worldwide [1,2]. While most DENV infections are inapparent or subclinical, approximately 25% result in clinical illness, ranging from a self-limited illness, known as dengue fever (DF), to more severe and potentially life-threatening disease, previously referred to as dengue hemorrhagic fever (DHF) and dengue shock syndrome (DSS) [1–3]. In 2009, the World Health Organization revised the clinical case definition to dengue, dengue with warning signs, and severe dengue [3].

Following primary DENV infection, individuals develop long-lived protection against the serotype that they are infected with. During secondary infection with a different DENV serotype, individuals have a higher risk of developing severe disease compared with those experiencing primary infection [1,4]. Considerable efforts have been made to develop therapeutic interventions, but no licensed antiviral drug is currently available [1]. While several candidate DENV vaccines are in different phases of clinical trials, only Dengvaxia, a chimeric yellow fever-dengue tetravalent DENV vaccine, has been licensed [5,6]. However, due to its low efficacy and increased risk of severe disease accompanying breakthrough DENV infection among DENV-naive individuals, Dengvaxia has been recommended only for DENV seropositive individuals aged 9–45 years [6–11]. Studies on DENV seroprevalence can be conducted to assess the potential efficacy of DENV vaccine candidates and to identify individuals who will benefit from being vaccinated. Moreover, DENV seroprevalence studies can improve our understanding of the transmission dynamics among individuals and in different locations, which can, in turn, aid in the development of intervention strategies.

**Fig 1:**
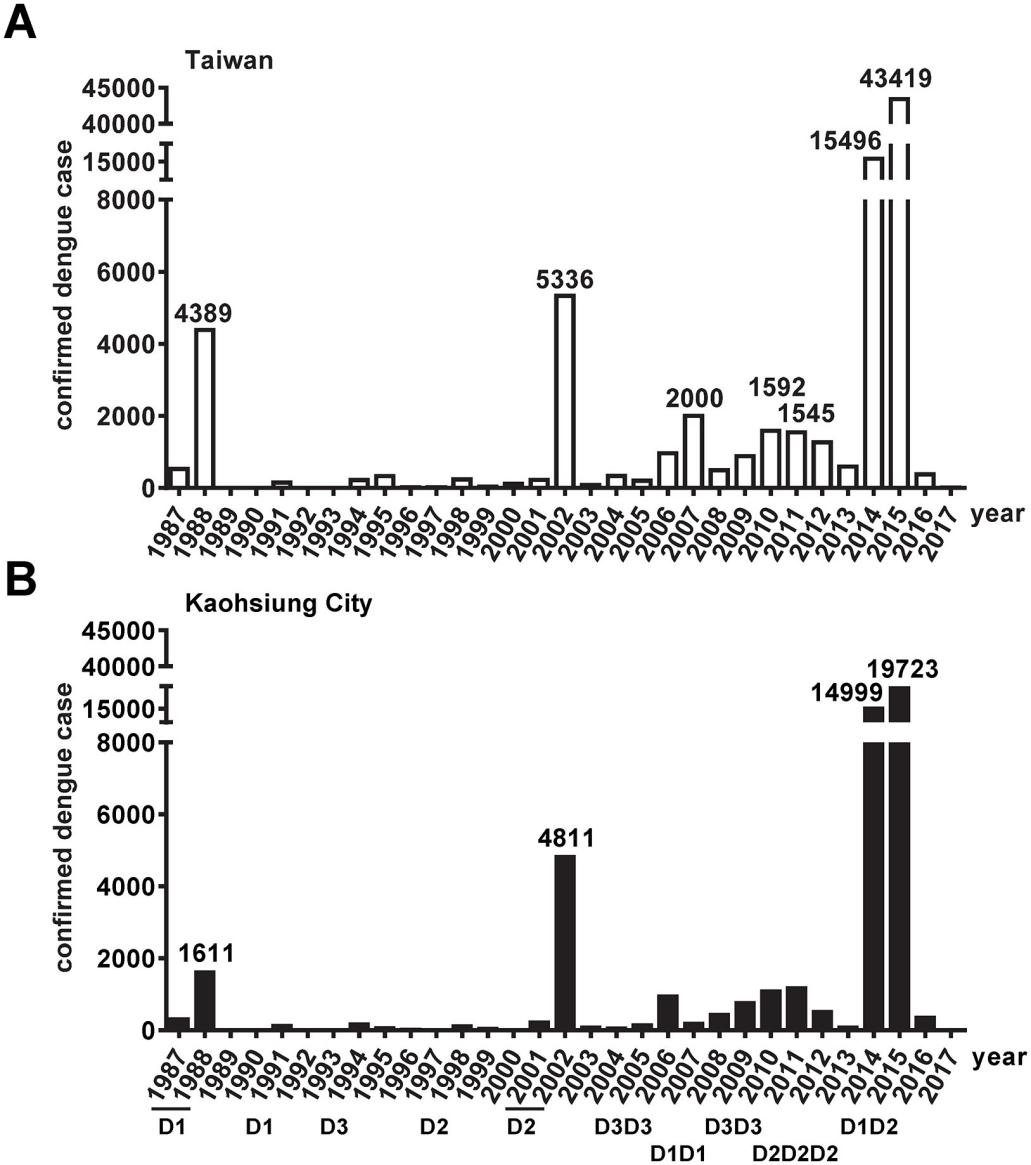
Dengue outbreaks in Taiwan from 1987 to 2017. Laboratory-confirmed indigenous dengue cases in Taiwan (A) and Kaohsiung City (B), based on data from CDC Taiwan [18]. Numbers of cases in major outbreaks (>1,500 cases) and the principal DENV serotype of each outbreak in Kaohsiung City are shown [13,16,18].

Epidemics of dengue disease in Taiwan have been documented since 1902 with an island-wide outbreak occurring in 1942–1943. After World War II (WWII), no dengue outbreak was reported for nearly four decades until 1981, when a DENV2 outbreak occurred in an off-shore islet, the Liuchiu township [12–14]. This was followed by a large DENV1 outbreak of >4,000 cases in Kaohsiung City and Pingtung county in 1987–1988. Since then, outbreaks of 100–300 confirmed DENV cases have occurred in southern Taiwan every 2 to 3 years until 2001–2002, when another large DENV2 outbreak of >5,000 confirmed cases took place [15] (Fig 1). Starting from 2004, a pattern of >200 cases per year as baseline and frequently >800 cases was observed until 2014, when an outbreak (primarily DENV1) with 15,492 confirmed cases occurred in Kaohsiung City. This was followed by another outbreak (primarily DENV2) with 43,419 confirmed cases mainly in Tainan and Kaohsiung Cities in 2015, which was the largest in Taiwan after WWII [16,17] (Fig 1). With few exceptions, DENV outbreaks have occurred exclusively in southern Taiwan, and Kaohsiung City has been most frequently affected.

Dengue is a notifiable communicable disease in Taiwan and a national web-based notifiable diseases surveillance system has been established since 1997. All confirmation laboratory tests are performed at the Centers for Disease Control (CDC), Ministry of Health and Welfare, Taiwan [18–20]. However, no seroprevalence study of dengue in Taiwan has been reported since 1989 [21]. Therefore, in this study, we conducted a DENV seroprevalence study in two districts of Kaohsiung City, one (Sanmin district) with high case numbers and the other (Nanzih district) with low case numbers during recent outbreaks.

## Methods

### Ethics statement and human sera

The study was approved by the Institutional Review Board (IRB) of the Kaohsiung Medical University Hospital (KMUH-IRB-960195 and KMUHIRB-(I)-20170185) with written informed consent from all adult participants (≥20 years old [y/o]) and from parents of those <20 y/o (of whom only 12 participants were <18 y/o). All serum samples were coded for anonymity and the analysis was approved by the IRB of the University of Hawaii at Manoa (CHS#17568).

**Fig 2:**
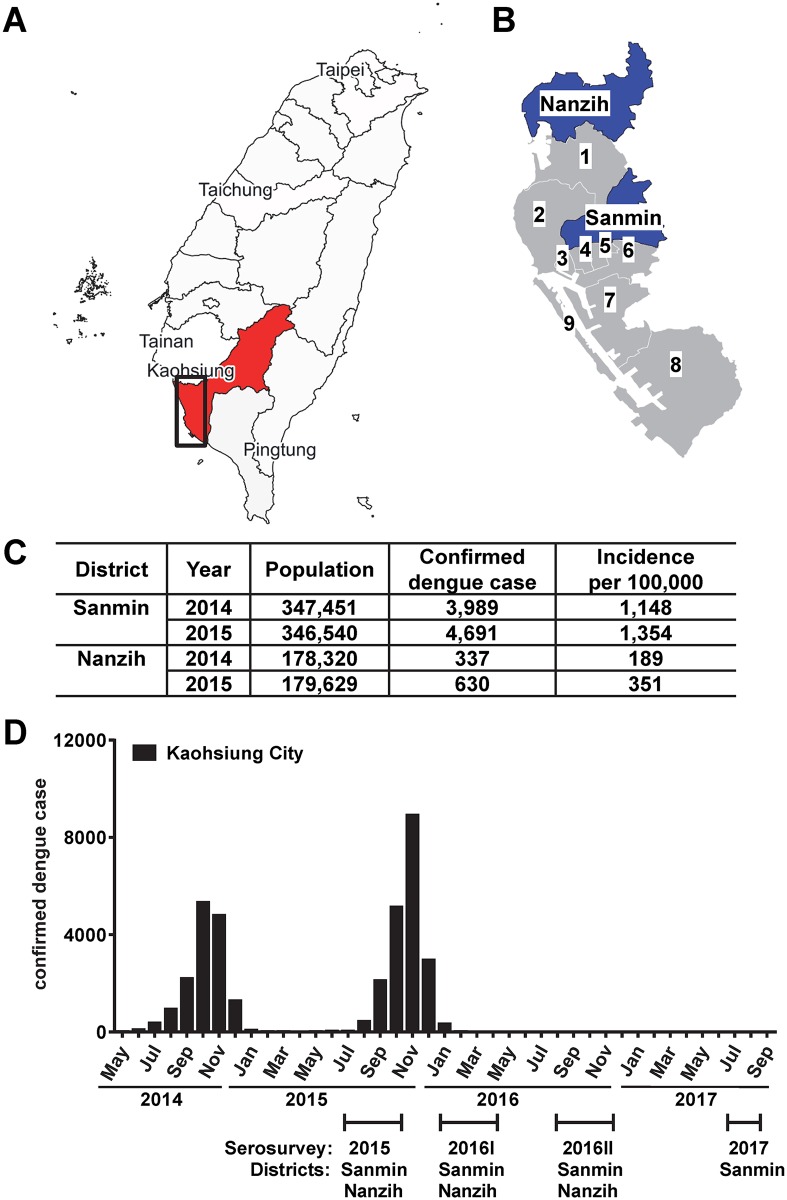
DENV seroprevalence survey in two districts of Kaohsiung City during and after the dengue outbreak in 2015. (A) Kaohsiung City is a metropolitan area in southern Taiwan. (B) Insert map of A: the Sanmin and Nanzih districts, together with other 9 districts, are located in the historic area of Kaohsiung City. (C) Population, confirmed indigenous dengue cases and incidence during the 2014 and 2015 outbreaks in the Sanmin and Nanzih districts [18,22]. (D) Study participants were enrolled at the beginning of the 2015 outbreak (2015) and during three periods after the outbreak (2016I, 2016II, and 2017). The numbers of dengue cases each month during the 2014 and 2015 outbreaks are presented [18]. Sources of maps: https://commons.wikimedia.org/wiki/File:KaohsiungCity_SanminDistrict.png and http://www.qgis.org/en/site/.

Kaohsiung City is a metropolitan area in southern Taiwan with a population of 2.8 million and consists of 38 districts [22]. The study enrolled participants from two districts (Sanmin and Nanzih) at the beginning of the 2015 outbreak (between August and November 2015) and during three periods after the outbreak (2016I: between February and May 2016; 2016II: between September 2016 and January 2017; and 2017: between August and September 2017) (Fig 2). From June 2016 to September 2017, only 5 indigenous dengue cases were confirmed in Kaohsiung City [18]. All residents in the two districts were invited to visit the study sites at different community activity centers (enrolling 111 to 215 participants per district during each survey). To record seroconversion, all participants in the 2015 survey were contacted to provide a second blood sample during the 2016I survey. All participants were asymptomatic at the time of enrollment and sample collection; 8 mL blood samples were collected through venipuncture, and sera were processed on the same day and stored at -80 °C until use. Questionnaires regarding demographic and socioeconomic information (such as age, sex, education, income), history of dengue, and history of chronic diseases including hypertension, diabetes, cardiovascular diseases, tuberculosis, cancer, chronic hepatitis B and C virus infection, and Japanese encephalitis virus (JEV) vaccination or infection were obtained. Overall, 9 (0.8%) and 31 (2.8%) of the 1,088 participants had a history of recent (2015) and past dengue, respectively; these included individuals with laboratory-confirmed, clinically suspected or self-reported dengue. Of the 417 participants in the 2015 survey, 127 (30.5%) returned voluntarily to provide a follow-up serum sample. Only one (0.8%) had a history of recent dengue, suggesting that individuals returning for re-testing were not doing so because of recent DENV infection.

### Serological tests

All serum samples (at 1:100 dilution) were tested by the InBios DENV detect IgG ELISA (DENV *Detect*IgG ELISA kit, InBios International, Inc.), which utilizes a recombinant DENV envelope (E) protein as antigen. The ratio of optical density (OD) to DENV-derived recombinant antigen and OD to negative control antigen was calculated as the immune status ratio (ISR). ISR of ≤1.65, ISR of 1.65–2.84 and ISR of ≥2.84 were interpreted as negative, equivocal and positive, respectively. Equivocal samples were repeated in duplicate to determine the immune status according to the manufacture’s instruction. The performance of the assay was validated by a previously described focus-reduction neutralization test (FRNT) and DENV inactivated-virion IgG ELISA in 105 samples with a sensitivity of 90% and specificity of 100% [23].

To identify recent infection during the 2015 DENV2 outbreak, an in-house DENV2-virion IgM ELISA was used for serum samples, which were positive or equivocal based on the InBios DENV IgG ELISA during the 2015 and 2016I surveys. Briefly, pellets of DENV2 virions derived from ultracentrifugation of culture supernatants of virus-infected Vero cells or pellets of mock-infected Vero cells were UV inactivated, diluted in coating buffer and loaded on flat-bottom 96-well plates at 4°C overnight, as described previously [23]. This was followed by blocking with 1% BSA in PBS for 1 h and incubation with serum samples (diluted 1:100), which had been preincubated with Gullsorb reagent (Meridian Bioscience) for 10 min to avoid interference of serum IgG or rheumatoid factor on IgM bound to antigen, followed by anti-human IgM conjugated to horseradish peroxidase, TMB substrate and stop solution [24–26]. Each ELISA plate included two positive controls (samples with confirmed DENV infection within 3 months post-infection) and four negative controls (flavivirus-naïve samples) all in duplicate, as described previously [15,23,24]. The OD at 450 nm was read with a reference wavelength of 650 nm; samples with signal greater than 3 standard deviations of the mean signal from four negative controls were considered positive [23].

A subset of the DENV2-IgM positive samples was further tested with a previously described FRNT: all neutralized DENV2 and/or DENV1, verifying our IgM assay [23]. Based on the pattern of FRNT of 13 IgM-positive samples, 8 were primary and 5 were secondary DENV infections [23].

### Statistical analysis

The two-tailed Fisher’s exact test was employed to compare categorical variables including seroprevalence rate, sex, and underlying diseases between two groups. The two-tailed unpaired t-test was used to compare continuous variables using GraphPad Prism 6.0. The 95% confidence interval (CI) was calculated in Excel. Spearman correlation test was used to determine the relationship between age-specific seroprevalence and proportion of severe dengue among total dengue cases using GraphPad Prism 6.0.

## Results

### DENV seroprevalence in two districts of Kaohsiung City

A total of 1,088 participants, primarily adults (98.9% ≥18 y/o), from two districts of Kaohsiung City were enrolled during and following the 2015 DENV outbreak (2015, 2016I, 2016II and 2017) (Fig 2). In Sanmin district, there were 627 participants (mean age, 37.6 y/o; range, 9–85 y/o; male to female ratio, 0.56). In Nanzih district, there were 461 participants (mean age, 45.6 y/o; range, 10–85 y/o; male to female ratio, 0.59). The age distribution of the study participants from each district was representative of residents in each district (S1 Table). There was no difference in the sex distribution between the two districts, but the participants from Sanmin were younger (P = 0.75 and <0.0001, Fisher’s exact test and unpaired t-test, respectively, two-tailed).

Testing with the InBios DENV detect IgG ELISA revealed 103 positive, 10 equivocal and 515 negative samples in Sanmin district, corresponding to an overall IgG seroprevalence rate of 16.4% (95% CI: 13.5–17.9%) (Table 1). During the 2016I survey right after the 2015 outbreak, 50 of the 202 participants (24.8%) enrolled in 2015 provided a second blood sample; one was found to seroconvert, corresponding to a seroconversion rate of 2% (95% CI: -1.9–4.0%). In Nanzih district, 32 samples were positive, 9 were equivocal and 420 were negative, corresponding to an overall IgG seroprevalence rate of 6.9% (95% CI: 4.6–8.1%). Seventy seven of the 215 participants (35.8%) enrolled in 2015 provided a second blood sample; one seroconverted, corresponding to a seroconversion rate of 1.3% (95% CI: -1.2–2.6%). Consistent with the higher number of dengue cases in Sanmin district compared with Nanzih district (3.8 fold higher) during the recent outbreaks (Fig 2C), the overall DENV seroprevalence rate in Sanmin district (16.4%) was 2.4 fold higher than that in Nanzih district (6.9%, P<0.0001, Fisher’s exact test, two-tailed). Combining data from the two districts, the overall DENV IgG seroprevalence was 12.4% (95% CI: 10.5–13.4%). There was no difference in the DENV seroprevalence rate between males and females (14.4% vs. 11.3%, P = 0.15, Fisher’s exact test, two-tailed).

**Table 1:** DENV seroprevalence rates in two districts of Kaohsiung City.

District	Survey	Primary sampling	Dengue IgG results ^a^			Repeat sampling in 2016I	Results
			positive	equivocal	negative		seroconversion ^b^
Sanmin	2015	202	33 (16.3%)	3 (1.5%)	166 (82.2%)	50	1 (2.0%)
	2016I	161	22 (13.7%)	5 (3.1%)	134 (83.2%)		
	2016II	111	19 (17.1%)	2 (1.8%)	90 (81.1%)		
	2017	153	28 (18.3%)	0 (0%)	125 (81.7%)		
	overall ^c^	628	103 (16.4%)	10 (1.6%)	515 (82.0%)		
Nanzih	2015	215	15 (7.0%)	1 (0.5%)	199 (92.5%)	77	1 (1.3%)
	2016I	131	12 (9.1%)	5 (3.8%)	114 (87.0%)		
	2016II	115	4 (3.5%)	3 (2.6%)	108 (93.9%)		
	overall ^c^	462	32 (6.9%)	9 (2.0%)	421 (91.1%)		

^a^ Results are presented as number (%) based on the InBios DENV detect IgG ELISA.

^b^ Seroconversion is defined by primary sampling negative and repeat sampling positive based on the InBios DENV detect IgG ELISA, and presented as number (%).

^c^ Overall includes the seroconverted sample in each district.

### Ratio of inapparent to symptomatic DENV infections

In addition to capturing seroconversion between the 2015 and 2016I surveys, we also tested samples using a DENV2-virion IgM ELISA during the 2015 (August to November) and 2016I (February to May) surveys to identify infection during the 2015 outbreak, which began in July 2015 and ended in January 2016. There were 19 IgM positive out of 363 samples in Sanmin district and 7 IgM positive out of 346 samples in Nanzih district. We used the number of IgM positive or seroconversion samples (Table 1) divided by the number of samples tested to determine the infection rate and total infection based on the population size (Table 2). In Sanmin district, the infection rate was 5.23% (95% CI: 2.94–6.40%); we estimated 18,124 total infections, including 4,691 symptomatic infections (corresponding to confirmed indigenous DENV cases,Fig 2C) and 13,433 inapparent infections with a ratio of inapparent to symptomatic infections of 2.86. Using a similar calculation, the infection rate was 2.02% (95% CI: 0.54–2.78%) in Nanzih district; we estimated 2,999 inapparent and 630 symptomatic infections, corresponding to a ratio of inapparent to symptomatic DENV infections of 4.76.

**Table 2:** Ratio of inapparent to symptomatic DENV infections during the 2015 outbreak in Kaohsiung City.

District	Population ^a^	Infection rate ^b^	Total infections ^c^	Symptomatic infections ^d^ (%)	Inapparent infections ^e^ (%)	Inapparent/symptomatic
Sanmin	346,540	5.23%	18,124	4,691 (25.9%)	13,433 (74.1%)	2.86
Nanzih	179,629	2.02%	3,629	630 (17.4%)	2,999 (82.6%)	4.76

^a^ Based on census data from Kaohsiung City [22].

^b^ Infection rate was estimated by the number of IgM-positive samples in the 2015 and 2016I surveys or seroconversion in the 2016I survey (Table 1) divided by the number of samples tested in each district.

^c^ Total infections were estimated by multiplying the population size by the infection rate.

^d^ Symptomatic infections corresponded to the number of confirmed indigenous dengue cases based on data from the Centers for Disease Control of Taiwan [18].

^e^ Inapparent infections were estimated by subtracting the number of symptomatic infections from the number of total infections.

### Age-specific DENV seroprevalence

**Fig 3:**
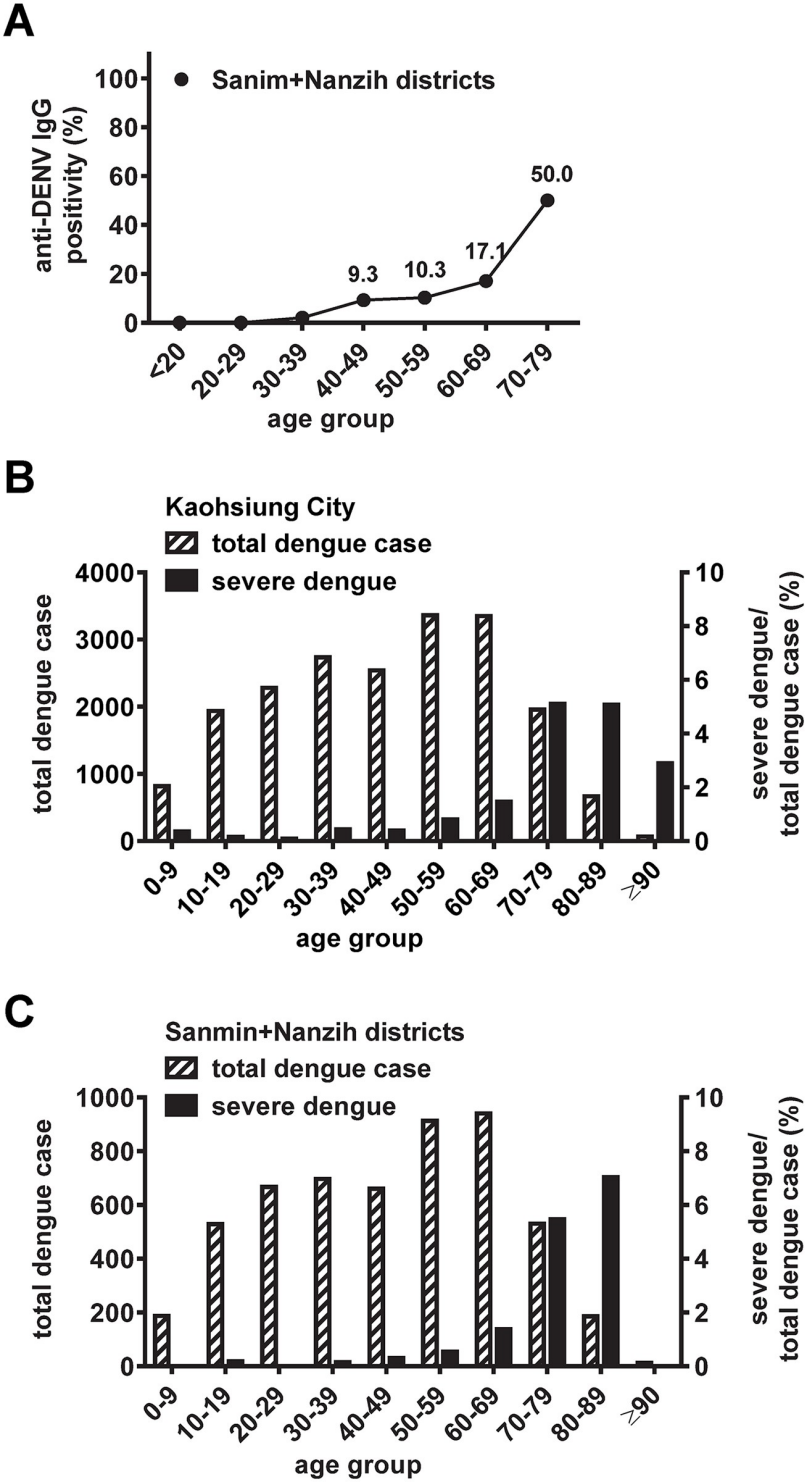
Age-specific DENV seroprevalence in two districts of Kaohsiung City in comparison with dengue cases and proportion of severe dengue. (A) DENV seroprevalence was based on the InBios DENV detect IgG ELISA; data in the 80–89 y/o group were not included due to the small number of participants. (B,C) The numbers of age-specific confirmed indigenous dengue cases and proportion of severe dengue among total dengue cases in each age group in Kaohsiung City (B) and in both Sanmin and Nanzih districts (C) were based on data from CDC Taiwan [18].

We further analyzed DENV seroprevalence stratified by age group (S2 Table). Based on the 2015 survey in Sanmin district, DENV seroprevalence rates increased with age from 2% (20–29 y/o), to 4.8% (30–39 y/o), 32.1% (60–69 y/o) and 62.9% (70–79 y/o). In Nanzih district, seroprevalence rates were 4.8% (20–49 y/o), 9.3% (60–69 y/o) and 36.4% (70–79 y/o). The age-specific DENV seroprevalence of the two districts is shown in Fig 3A. Despite the difference between the two districts, a sharp and significant increase in seroprevalence from the 50–69 y/o group (born between 1946 and 1965) to those ≥70 y/o (born before 1945) was observed in both districts (P = 0.0002 and 0.002 for Sanmin and Nanzih districts, respectively, Fisher’s exact test, two-tailed), suggesting that the historical island-wide outbreak of 1943–1944 may account for the higher seroprevalence rate among the elderly (≥70 y/o) in Kaohsiung City.

**Fig 4:**
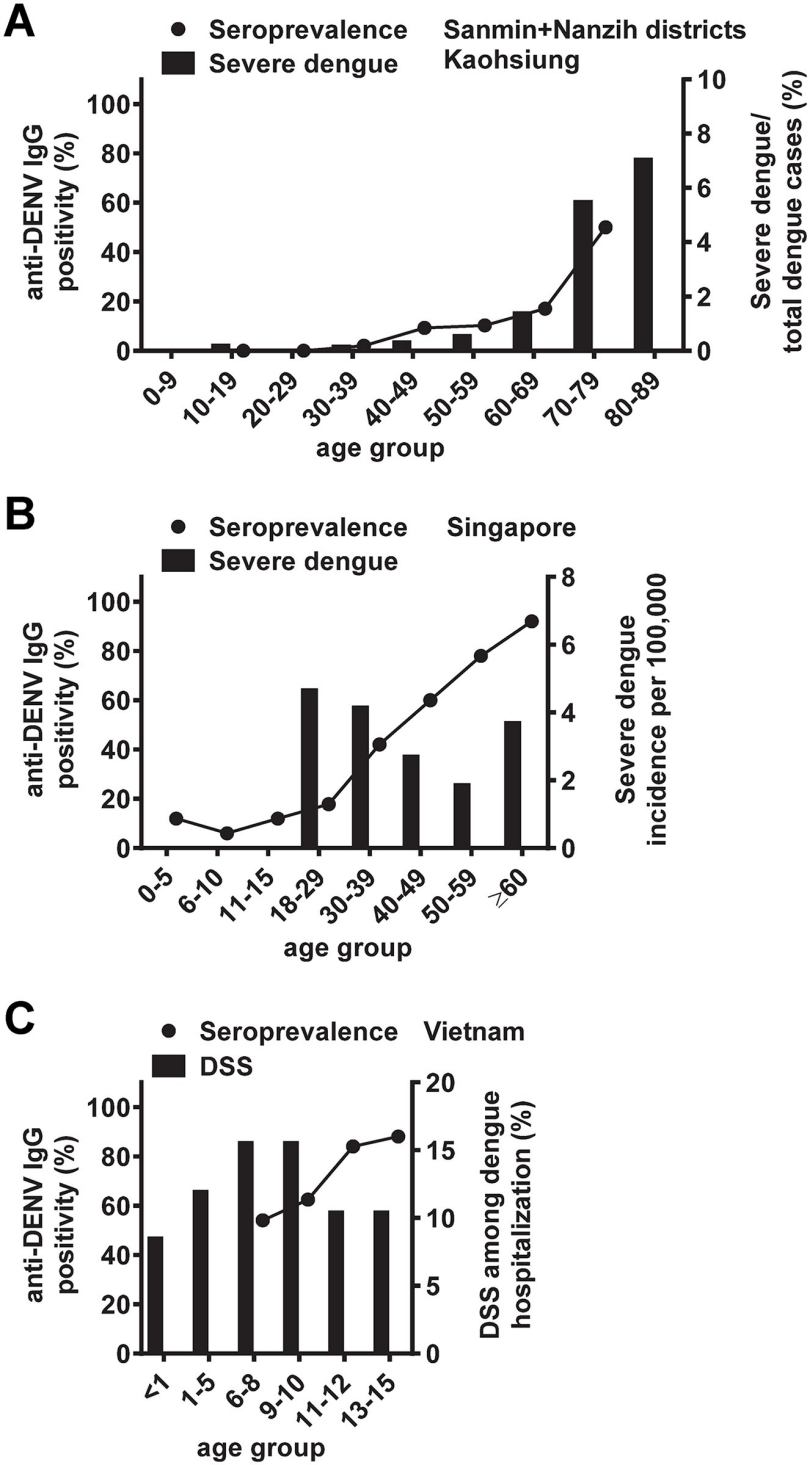
Relationship between age-specific DENV seroprevalence and clinically severe dengue disease in different countries. (A) Age-specific DENV seroprevalence and proportion of severe dengue among total dengue cases in each age group in Sanmin and Nanzih districts of Kaohsiung City based on this study. (B) Age-specific DENV seroprevalence and incidence of severe dengue in Singapore, based on previous studies conducted between 2005 and 2010 [33,52,53]. (C) Age-specific DENV seroprevalence in school children and proportion of DSS among pediatric dengue hospitalizations in southern Vietnam, based on previous studies conducted between 2001 and 2009 [46,51].

The 2015 dengue outbreak in Kaohsiung City resulted in 19,723 confirmed dengue cases (S3 Table). The distribution of dengue cases increased gradually with age and peaked in the 50–69 y/o group (Fig 3B). The proportion of severe dengue among total dengue cases started to increase in the 50–59 y/o group and peaked in the 70–79 and 80–89 y/o groups (Fig 3B); similar trends were observed based on the number or incidence of severe dengue in different age groups (S1 Fig). The age-specific dengue cases and proportion of severe dengue in both the Sanmin and Nanzih districts showed a pattern similar to that of Kaohsiung City (Fig 3C). Interestingly, the increase in the proportion of severe dengue with age correlated well with the increase in DENV seroprevalence with age (Spearman correlation coefficient r = 0.94, P = 0.005, two-tailed Spearman correlation test) (Fig 4A).

### JEV vaccination and DENV IgG positivity

A nationwide JEV vaccination campaign in Taiwan was launched in the 1960s; children <3 y/o received two doses of inactivated JEV vaccine initially, followed by an increase to three doses (with a booster dose after one year) since 1974 and to four doses (with the last dose during the first year of elementary school) since 1976 [27]. The coverage rates were reported to be >80% and 95% for the two-/three-dose and the four-dose schedules, respectively, whereas people born before 1963 have never been vaccinated [28,29]. Due to the cross-reactivity of anti-E antibodies with different flaviviral E proteins, there were concerns about the cross-reactivity induced by JEV vaccination on DENV serological tests [30–32]. To investigate whether JEV vaccination affected our estimates of DENV seroprevalence, we analyzed the relationship between DENV IgG positivity and history of JEV vaccination or JEV infection through questionnaires during the 2017 survey. Study participants ≤54 y/o (born in 1963 or after) were considered to have received JEV vaccine, and those who were >54 y/o and responded “yes” on questionnaires were considered to have experienced previous JEV infection. As shown in Table 3, 46% of those with DENV IgG positivity had JEV vaccination or infection, which was significantly lower than the 78% of those with DENV IgG negativity (*P* = 0.0017, Fisher’s exact test, two tailed), suggesting that DENV IgG positivity among adults in this study was unlikely attributed to previous immunization with inactivated JEV vaccine >14 years before during their childhood.

**Table 3:** DENV IgG positivity and history of JEV vaccination or infection.

	JEV vaccination or infection ^b^					
	≤54 y/o			>54 y/o		
DENV IgG results ^a^	yes ^b^	no	unknown	yes ^c^	no	unknown
positive (n = 28)	13	0	0	0	9	6
equivocal (n = 0)	0	0	0	0	0	0
negative (n = 125)	92	0	0	6	18	9

^a^ Based on 153 participants during the 2017 survey using the InBios DENV detect IgG ELISA.

^b^ Nationwide JEV vaccination campaign in Taiwan started in the 1960s. Participants ≤54 y/o (born in 1963 or after) were considered to have received JEV vaccine.

^c^ Participants >54 y/o (born before 1963) were considered to have never been vaccinated, and “yes” indicated history of previous JEV infection.

### Underlying diseases and DENV IgG positivity

Several underlying diseases such as diabetes, hypertension, cardiac disease and asthma have been associated with severe dengue or DHF/DSS among adults [33–38]. Based on questionnaires, we found the proportion of chronic diseases among DENV IgG positive individuals to be higher than that among DENV IgG negative individuals (30.4% vs 17.1%, *P* = 0.0003, Fisher’s exact test, two tailed), including hypertension and diabetes (14.1% vs 6.0% and 5.2% vs 1.7%, *P* = 0.0006 and 0.01, respectively, Fisher’s exact test, two tailed). To exclude the confounding effect of age, we compared the <60 y/o and ≥60 y/o groups and found that the proportion of hypertension among those <60 y/o was higher for those with DENV IgG positivity compared with those with DENV IgG negativity (S4 Table).

## Discussion

In this study, we investigated DENV seroprevalence in a major city in southern Taiwan affected by dengue outbreaks for decades. Our findings on the heterogeneity of DENV seroprevalence in a metropolitan area and a pattern of age-specific DENV seroprevalence which differed from that of DENV-hyperendemic countries have implications for the use of currently available, as well as future, DENV vaccines in countries of low DENV endemicity and for understanding the epidemiology of severe dengue in these countries.

The overall DENV seroprevalence in Kaohsiung City was 12.4%. Despite the low seroconversion rate, we used a DENV2-virion IgM ELISA to identify recent infections and calculated the infection rate of the 2015 outbreak. Due to the transient nature of IgM production (duration of ~3–4 months) and the gap between the 2015 and 2016I surveys (Fig 2D), the possibility of underestimating the infection rate cannot be completely ruled out. Nonetheless, we found that the infection rate in Sanmin district was higher than in Nanzih district (Table 2); consistent with this, a mosquito survey in September 2015 showed higher Breteau, house, larva and adult indices in Sanmin district compared with Nanzih district [18]. Sanmin is a heavily populated district with many small parks used for community activities, and many new buildings under construction, where unattended waste and water accumulation with high mosquito density have been reported by the city. In contrast, Nanzih is a less populated industrial district with factories and unused land [22]. The observation that DENV seroprevalence in Sanmin district (16.4%) is higher than that in Nanzih district (6.9%) suggests district-level differences in a metropolitan area and highlights the importance of checking the DENV immune status of each individual before Dengvaxia vaccination [9–11].

We further calculated the total, symptomatic, and inapparent infections, and the ratios of inapparent to symptomatic infections (2.86 and 4.76) were within the range that has been reported in the literature [39–44]. With the implementation of both passive and active surveillance systems in Taiwan, imported and indigenous confirmed dengue cases have been tested at the CDC Taiwan [18–20]. Cumulatively, confirmed indigenous dengue cases in Kaohsiung City since 1981 were 47,882 [18]; assuming that the proportion of symptomatic infections ranges from 5% to 40% [39–44], the number of total infections was estimated to be from 119,705 to 957,640, corresponding to a DENV seroprevalence rate ranging from 4.3% to 34.5%. Our observed dengue seroprevalence of 12.4% (95% CI: 10.5–13.4%) was within this range.

In agreement with reports from other DENV-endemic regions, DENV seroprevalence in Kaohsiung city increased with age. However, the patterns of age-specific DENV seroprevalence and peak of clinically severe disease among the elderly were different from those in the Americas and Southeast Asia, where dengue hospitalization peaked in children or young adults [45–48]. In Brazil, a study in 2006 reported that the DENV seroprevalence rate increased from 54% (5–6 y/o group) to 85% (10–14 y/o group), and the mode of monotypic immunes, which corresponds to the peak of severe disease in age, was estimated to be 14 y/o [45]. Consistent with this, the peak age of DHF cases shifted from 20–40 y/o before 2007 to <15 y/o after 2007 [45,49,50]. In southern Vietnam, the DENV seroprevalence rate increased from 54% (7–8 y/o group) to 84% (11–12 y/o group), and the peak of proportion of DSS among cases of pediatric dengue hospitalization was in the 6–10 y/o group (Fig 4C) [46,51]. In Singapore, the DENV seroprevalence rate increased from 42% (30–49 y/o group) to 78% (50–59 y/o group); the incidence of severe dengue showed two peaks: one in the 18–29 y/o group probably representing the monotypically immune population and the other in the ≥60 y/o group probably associated with underlying diseases among the elderly (Fig 4B) [33–35,52,53].

Based on the 2015 survey, the DENV seroprevalence rate in Sanmin and Nanzih districts increased from 2.1% (30–39 y/o), to 17.1% (60–69 y/o) and 50% (70–79 y/o) (Fig 4A). The sharp increase in DENV seroprevalence in the 70–79 y/o group paralleled a drastic increase in the proportion of severe dengue among total dengue cases in that age group, suggesting that in addition to age and other risk factors, significant numbers of monotypically immune individuals in that age group may contribute to the observed severe dengue diseases among the elderly in Taiwan [14,36–38]. In agreement with this, analysis of indigenous dengue cases from 2002 to 2007 revealed that the majority of DHF cases (84% of total DHF cases or DHF cases ≥60 y/o) were secondary DENV infections, whereas the majority of DF cases were primary DENV infections except in 2002 [14]. We have performed FRNT to DENV1 and DENV2 for 20 DENV IgG positive samples from the elderly (60–69 and 70–79 y/o age groups) in the Sanmin district and found 40% and 60% to be monotypically and multitypically DENV-immune, respectively. After an island-wide outbreak presumably of DENV1 during WWII (1942–1943), there have been several DENV2 outbreaks in Kaohsiung City (2001–2002, 2003, 2011, 2012 and 2013) before 2015 (Fig 1B), making the assessment of the time-interval between DENV1 and DENV2 infections more complicated. Based on the DENV seroprevalence among those ≥70 y/o during the 2015 survey (52.8%) and the assumption that they were infected during the 1942–1943 outbreak, the attack rate was estimated to be 52.8%.

The observation that the proportion of hypertension among participants <60 y/o was higher for DENV IgG positive than DENV IgG negative subjects (S4 Table) suggest that certain risk factor might associate with DENV IgG positivity or susceptibility to DENV infection. Growing evidence supports the role of the gut microbiota in the pathogenesis of hypertension and perturbation of the gut microbiota has been reported to increase the susceptibility to DENV and other flavivirus infections in mice, though the mechanisms remain to be explored in future studies [54,55]. It is worth noting that the proportion of comorbidities among DENV IgG positive subjects (41/131 = 30.4%) or among all participants (209/1090 = 19.2%) was lower than the proportion of comorbidities (hypertension, diabetes and cardiovascular diseases together) for adults ≥18 y/o in Taiwan (41.8%) [56], suggesting that a bias of enrolling individuals with comorbidities was unlikely, though a bias of enrolling those with fewer comorbidities cannot be ruled out.

As described in the Methods section, all study participants were asymptomatic during enrollment and only 0.8% of the 1,088 participants and 127 returning participants had a history of recent dengue, suggesting that the enrollment or follow-up was unlikely to be biased by a history of recent dengue. In addition, our questionnaires revealed that 79 (7.3%) of the 1,088 participants had a history of dengue among family members (though not limited to those in the same household), which was lower than the DENV seroprevalence of 12.4% of this study but higher than the estimate of dengue prevalence in Kaohsiung City (1.7%) based on cumulative confirmed cases [18]. Thus, a potential bias of enrolling those with dengue among family members cannot be completely ruled out.

There are several limitations to our study. First, it is worth noting that many commercially available IgG kits, some of which capture human IgG first followed by adding DENV antigens and detecting reagents, have been calibrated to detect high-titer IgG and, therefore, are more suitable to test for recent DENV exposure. The possibility that the InBios DENV detect IgG ELISA has lower sensitivity for detecting remote DENV infections cannot be completely ruled out. However, this caveat is unlikely to affect our major finding of a sharp and significant increase in DENV seroprevalence in the 70–79 y/o age group. Second, future studies involving larger sample sizes, randomicity and other districts are crucial to verify the observations in this study. Third, while this study suggests that DENV seroprevalence is low in children (7.1%) based on limited numbers of participants <20 y/o, future studies are needed to investigate the DENV seroprevalence among children, which will reflect interim dengue endemicities. Fourth, another limitation is that all blood samples during the 2015 survey were not taken prior to onset of the 2015 outbreak (Fig 2). Notably, of the 127 repeated samples three were DENV IgM positive during the 2015 survey. A study of the 1997 outbreak in Santiago de Cuba also reported a low primary DENV infection rate (2%), probably related to focal transmission and heterogeneous distribution of *A*. *aegypti* [57]. Additionally, although we found that DENV IgG positivity was unlikely to be attributable to immunization with inactivated JEV vaccine >14 years before, future studies involving sequential samples from recipients of different JEV vaccines (inactivated and live-attenuated vaccines) are needed to better understand the effect of JEV vaccination on DENV IgG serological tests.

## Supporting information

S1 Fig Age-specific dengue cases, severe dengue and incidence in Kaohsiung City and in two districts. (A) Relationship between age-specific dengue cases and severe dengue in Kaohsiung City and Sanmin and Nanzih districts combined. (B) Relationship between age-specific dengue cases and incidence of severe dengue in Kaohsiung City and the two districts. The numbers of confirmed indigenous dengue cases and incidence were based on data from CDC Taiwan and Kaohsiung City [20,22].
(TIF)

S1 Table Age distribution of study participants compared with that of the population in Kaohsiung City and in Sanmin and Nanzih districts. (XLSX)

S2 Table Age-specific DENV seroprevalence rates in two districts of Kaohsiung City. (XLSX)

S3 Table Age distribution of dengue and severe dengue cases during the 2015 outbreak in Kaohsiung City and in Sanmin and Nanzih districts. (XLSX)

S4 Table DENV IgG positivity and underlying chronic diseases among those <60 y/o. (XLSX)

S1 Checklist STROBE checklist. (DOC)
