# Supplementary figures and images for: Seroprevalence of dengue virus in two districts of Kaohsiung City after the largest dengue outbreak in Taiwan since World War II
Source: PLoS Negl Trop Dis. 2018 Oct 24;12(10):e0006879. doi: 10.1371/journal.pntd.0006879 (PMC6218099; doi:10.1371/journal.pntd.0006879)

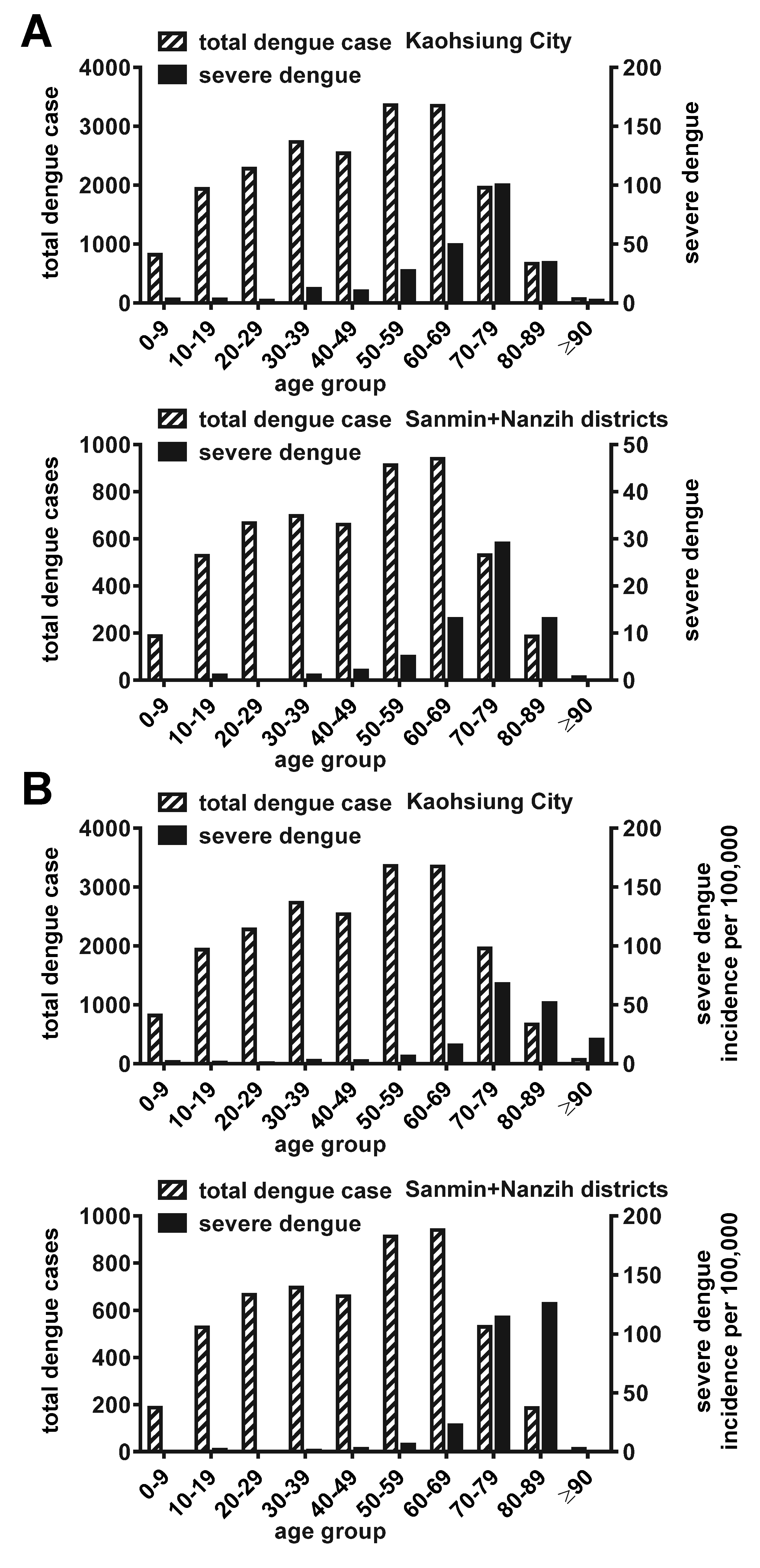

Supplement: S1 Fig — (A) Relationship between age-specific dengue cases and severe dengue in Kaohsiung City and Sanmin and Nanzih districts combined. (B) Relationship between age-specific dengue cases and incidence of severe dengue in Kaohsiung City and the two districts. The numbers of confirmed indigenous dengue cases and incidence were based on data from CDC Taiwan and Kaohsiung City [20,22]. (TIF) [file pntd.0006879.s001.tif]
